# Prediction of Phenotype-Associated Genes via a Cellular Network Approach: A *Candida albicans* Infection Case Study

**DOI:** 10.1371/journal.pone.0035339

**Published:** 2012-04-11

**Authors:** Yu-Chao Wang, Shin-Hao Huang, Chung-Yu Lan, Bor-Sen Chen

**Affiliations:** 1 Laboratory of Control and Systems Biology, Department of Electrical Engineering, National Tsing Hua University, Hsinchu, Taiwan; 2 Department of Life Science, National Tsing Hua University, Hsinchu, Taiwan; 3 Institute of Molecular and Cellular Biology, National Tsing Hua University, Hsinchu, Taiwan; Semmelweis University, Hungary

## Abstract

*Candida albicans* is the most prevalent opportunistic fungal pathogen in humans causing superficial and serious systemic infections. The infection process can be divided into three stages: adhesion, invasion, and host cell damage. To enhance our understanding of these *C. albicans* infection stages, this study aimed to predict phenotype-associated genes involved during these three infection stages and their roles in *C. albicans*–host interactions. In light of the principles that proteins that lie closer to one another in a protein interaction network are more likely to have similar functions, and that genes regulated by the same transcription factors tend to have similar functions, a cellular network approach was proposed to predict the phenotype-associated genes in this study. A total of 4, 12, and 3 genes were predicted as adhesion-, invasion-, and damage-associated genes during *C. albicans* infection, respectively. These predicted genes highlight the facts that cell surface components are critical for cell adhesion, and that morphogenesis is crucial for cell invasion. In addition, they provide targets for further investigations into the mechanisms of the three *C. albicans* infection stages. These results give insights into the responses elicited in *C. albicans* during interaction with the host, possibly instrumental in identifying novel therapies to treat *C. albicans* infection.

## Introduction

*Candida albicans* is the most prevalent opportunistic fungal pathogen in humans. It can cause superficial infections in the oral and vaginal mucosa as well as life-threatening systemic infections [1]. Because *C. albicans* is one of the leading causes of hospital-acquired bloodstream infections, a number of its virulence factors have been studied, including the ability to undergo morphogenesis and phenotypic switching, as well as the secretion of adhesins and hydrolytic enzymes [2]–[4]. In addition, *in vitro* infection models have demonstrated that the infection process can be divided into three stages: adhesion, invasion, and damage [5], [6]. The initial stage of host-pathogen interaction is characterized by the physical attachment of *C. albicans* to host tissues. In the subsequent stage, *C. albicans* enters host cells by active penetration and induced endocytosis [7], [8], known as the invasion stage. The last stage is characterized by substantial cell damage and destruction of host tissues [5]. Wächtler *et al.* used a systematic approach to examine the contribution of 26 selected genes and their mechanistic roles during the three infection stages [6]. Using specific gene deletion mutants, Wächtler *et al.* assessed the ability of each mutant to adhere to, invade, and cause damage in host cells. Although they successfully determined the extent of the contribution of each gene in the adhesion, invasion, and damage phenotypes during *C. albicans* infection, the construction of mutant strains is labor-intensive and time-consuming. Therefore, we aimed to predict phenotype-associated genes whose molecular functions may be responsible for the phenotypes of three infection stages in order to enhance our understanding of *C. albicans* infection.

The understanding of cells or organisms at the systems level has been a recurrent theme in the biological sciences [9]. Although the study of individual genes and proteins remains important, understanding the structure and dynamics of a biological system has become increasingly necessary. An organism is not simply an assembly of genes and proteins, but also the network properties and interconnectivity between genes and proteins, and these, in fact, facilitate the full functionality of a cell [10], [11]. Therefore, key research approaches are using experimental, statistical, and mathematical modeling to investigate these biomolecular networks to discover the basic function of genes and essential mechanisms involved in various biological phenomena [12]. Many studies have deduced the functions of various proteins using protein-protein interaction (PPI) networks [13]–[15]. Nevertheless, the protein interaction data collected from published literature and databases include results obtained under different experimental conditions, which may be inappropriate for the characterization of *C. albicans*-host interaction during the infection process, specifically. Recently, we developed a mathematical scheme to construct gene regulatory and protein interaction networks based on the integration of omics data [16]. The proposed method has been shown to be powerful and widely applicable, usable under different conditions and for different species. In this study, with the help of high-throughput omics data, network construction schemes were employed to construct the cellular networks, i.e., gene regulatory and protein interaction networks, involved in *C. albicans* infection and to predict the infection stage-associated genes in *C. albicans*.

**Figure 1:**
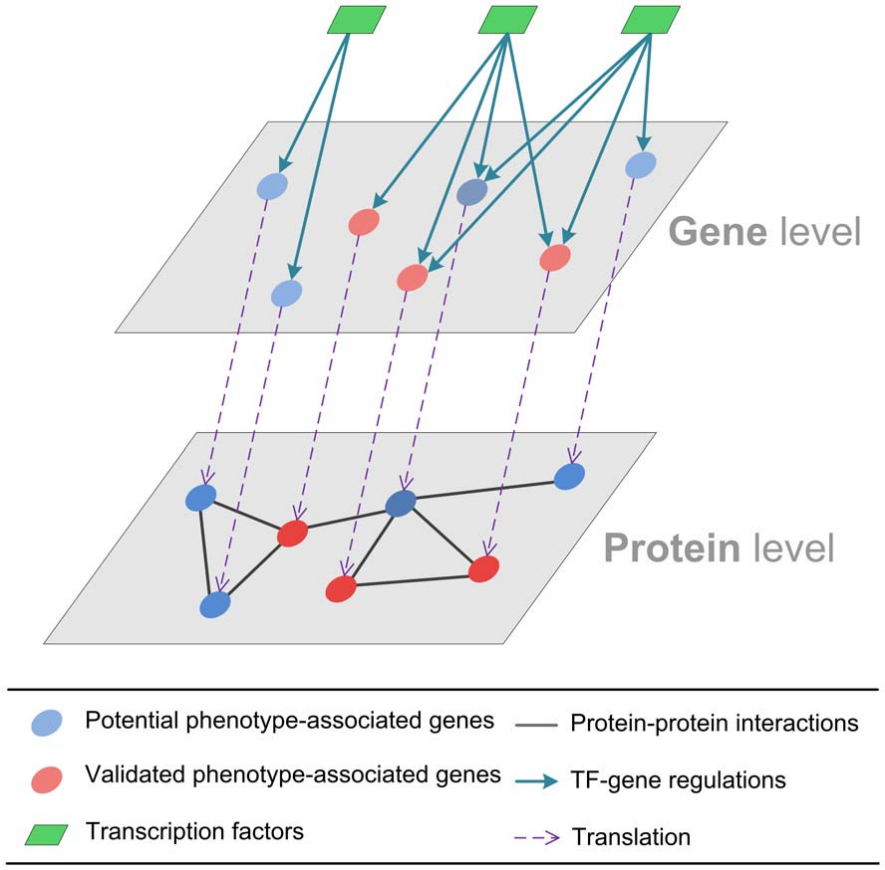
Schematic diagram for phenotype-associated gene prediction using cellular network approach. In light of the constructed gene regulatory network and protein interaction network, the predicted phenotype-associated genes are regulated by similar TFs that regulate experimentally validated genes at the gene level, and encode proteins that interact with many experimentally validated proteins at the protein level. The phenotype-associated gene and protein predicted by the cellular network approach is filled with crossed lines in the diagram.

The underlying principle of phenotype-associated gene prediction using cellular networks is that the proteins which lie closer to one another in a PPI network are more likely to have similar function [14]. Additionally, genes regulated by the same transcription factors (TFs) tend to have similar functions [17]. Consequently, these concepts are integrated and utilized to predict phenotype-associated genes based on gene regulatory and protein interaction networks as shown in Figure 1. In this model, some experimentally validated genes whose mutation result in defective adhesion, invasion, and damage phenotypes were first collected. Then, the predicted phenotype-associated genes are regulated by similar TFs that have been found to regulate experimentally validated genes at the gene level, and encode proteins which interact with many experimentally validated proteins at the protein level. In this study, the phenotype-associated genes responsible for the adhesion, invasion, and damage stages of infection were determined and their roles in *C. albicans*-host interactions were further investigated. These results provide insights into the responses of *C. albicans* upon its interactions with the host, possibly facilitating the development of new strategies to prevent and control *C. albicans* infection.

## Methods

### Method Overview and Data Selection

The method used to predict *C. albicans* infection stage-associated genes using cellular networks can be divided into two steps. 1. Construct *C. albicans* gene regulatory and protein interaction networks during infection. 2. Identify the genes regulated by similar TFs that regulate experimentally validated genes at the gene level, and encode proteins that interact with many experimentally validated proteins at the protein level based on the constructed cellular networks (Figure 1).

For the first step of cellular network construction, *C. albicans* TF-gene regulatory associations, *C. albicans* protein-protein interactions, and gene expression profiles during infection were required. However, high-throughput screening such as PPI and ChIP-chip data for *C. albicans* is currently limited. According to the facts that *C. albicans* and *Saccharomyces cerevisiae*, the most well-studied eukaryotic model organism [18], [19], are closely related (both fall within the hemiascomycete group), and that the *C. albicans* genome sequence is now available, allowing for the identification of orthologs between these two species, potential TF-gene regulatory associations and PPIs in *C. albicans* were inferred from the corresponding information in *S. cerevisiae* using ortholog information [20]. Regulatory associations between TFs and genes in *S. cerevisiae* were obtained from YEASTRACT database (http://www.yeastract.com/) [21]; protein-protein interaction data in *S. cerevisiae* were extracted from the Biological General Repository for Interaction Datasets (BioGRID) database (http://thebiogrid.org/) [22]; ortholog information between *C. albicans* and *S. cerevisiae* genes was acquired from the *Candida* Genome Database (CGD) (http://www.candidagenome.org/) [23]. If there was a regulatory association between TF A and Gene B in *S. cerevisiae* and TF A and Gene B had orthologs in *C. albicans* (TF A’ and Gene B’, respectively), we inferred that TF A’ potentially regulates Gene B’ in *C. albicans*, i.e., a potential TF-gene regulatory association exists between TF A’ and Gene B’ in *C. albicans* [20]. Potential protein-protein interactions in *C. albicans* could be inferred in a similar way. The inferred TF-gene associations and the currently available ChIP-chip information for *C. albicans* from published literature [24]–[38] could then be used for further analysis. In addition, genome-wide microarray data from Zakikhany *et al.* [5], which profiled time-course gene expression during an experimental *C. albicans* infection in reconstructed human oral epithelium (RHE) over 24 hours (1, 3, 6, 12, 24 hours post-infection with two to five biological replicates), were used in this study. RHE is a three-dimensional organotypic epithelial model of human oral and vaginal mucosa developed by SkinEthic Laboratories (France). As this model expresses all natural major markers of the epithelial basement membrane and epithelial differentiation, and even possesses tissue repair mechanisms, it was utilized to mimic *in vivo C. albicans* infection [39]. For the second step of infection stage-associated gene prediction, mutant phenotype data from the CGD [23] and published literature were employed to tally the experimentally validated genes involved in the adhesion, invasion, and damage stages of infection. Subsequently, three pools of infection stage-associated genes were created and used as the starting point for phenotype-associated gene prediction.

### Cellular Network Construction

The strategy for cellular network construction is to build candidate networks based on TF-gene regulatory associations/PPIs under all possible experimental conditions as reported in the literature and databases, and then to refine the candidate networks for a specific condition with the help of microarray data [16]. In light of all possible TF-gene regulatory associations/PPIs in *S. cerevisiae* and the ortholog information between *C. albicans* and *S. cerevisiae* genes, we can infer potential TF-gene regulatory associations/PPIs in *C. albicans* [20]. Consequently, the candidate gene regulatory network of *C. albicans* can be easily constructed by linking TFs and genes with potential TF-gene regulatory associations. Similarly, the candidate *C. albicans* protein interaction network can be constructed by linking proteins that potentially interact with each other.

Since the candidate gene regulatory and protein interaction networks were constructed using data obtained from literature and various databases where experiments were performed under different conditions, they may not appropriately represent the specific cellular process of interest during *C. albicans* infection. Therefore, it would be indeed tempting to refine these candidate networks using microarray data of RHE infection with *C. albicans*. In this study, dynamic models were employed to describe the dynamic transcriptional regulations between TFs and their target genes in the candidate gene regulatory network as well as dynamic interactions between proteins in the candidate protein interaction network [16] (see Text S1 for details). With the help of time-course microarray data, the system parameter estimation method and the model selection measurement Akaike Information Criterion (AIC) were then used to detect significant regulations and interactions in the cellular networks for *C. albicans* infection [16], [40], [41] (see Text S1 for details). As a result, the candidate cellular networks were refined and the gene regulatory and protein interaction networks for *C. albicans* infection were constructed.

### Infection Stage-associated Gene Prediction

In light of mutant phenotype information from the CGD [23] and literature evidence, three pools of experimentally validated genes involved in the adhesion, invasion, and damage stages of infection were specified and used as the starting point for *C. albicans* infection stage-associated gene prediction. Based on the constructed gene regulatory and protein interaction networks in *C. albicans* infection and the experimentally validated genes within, we aimed to find the genes with similar TFs and interacting translated proteins as described previously (Figure 1).

For each infection stage-associated gene pool, we first identified the significant TFs which regulate these experimentally validated genes [20] and then determined the potential infection stage-associated genes that are regulated by the significant TFs according to the constructed gene regulatory network. In this way, the potential infection stage-associated genes are regulated by similar TFs that regulate experimentally validated genes. For each TF in the constructed gene regulatory network, the quantity of regulations on the experimentally validated genes can be calculated and an empirical *p*-value can be computed to specify whether this TF significantly regulate those experimentally validated genes. If a TF regulates more experimentally validated genes in the constructed gene regulatory network, it is a more significant TF for the specific infection stage. To determine the empirical *p*-value for the observed quantity of regulations of a TF, a null distribution was generated by repeatedly permuting the network structure of the candidate gene regulatory network and computing the number of regulations on the experimentally validated genes for each random network structure. The network structure permutation was performed while keeping the network size constant, i.e., the target genes that a particular TF regulated were permuted without changing the total quantity of TF-gene regulatory associations of the network. The process was repeated 100,000 times and the empirical *p*-value for the observed quantity of regulations was estimated as the fraction of random network structures in which quantity of regulations on the experimentally validated genes of the specific TF was at least as large as the quantity of regulations in the real network structure [20]. The quantities of regulations with *p*-value ≤ 0.05 were determined as significant and the corresponding TFs were identified as the significant TFs for particular infection stages. Following from the significant TFs for each infection stage, the potential infection stage-associated genes were also identified as the ones that are regulated by most of those significant TFs (*p*-value ≤ 0.05).

For the constructed protein interaction network for *C. albicans* infection, we then identified whether or not the translated proteins of potential infection stage-associated genes lie closer to those proteins that have been experimentally validated. With a similar approach to the permutation of gene regulatory network structure, empirical *p*-values of the quantities of interactions on the experimentally validated proteins were computed for each potential infection stage-associated protein. The proteins with *p*-value ≤ 0.05 were determined to interact with many experimentally validated proteins in the PPI network and the corresponding genes were predicted as infection stage-associated genes. Therefore, starting from experimentally validated genes associated with *C. albicans* infection stages we could predict more genes that may be involved in the mechanism responsible for the adhesion, invasion, and damage phenotypes.

## Results

### Prediction of *C. albicans* Infection Stage-associated Genes

**Figure 2:**
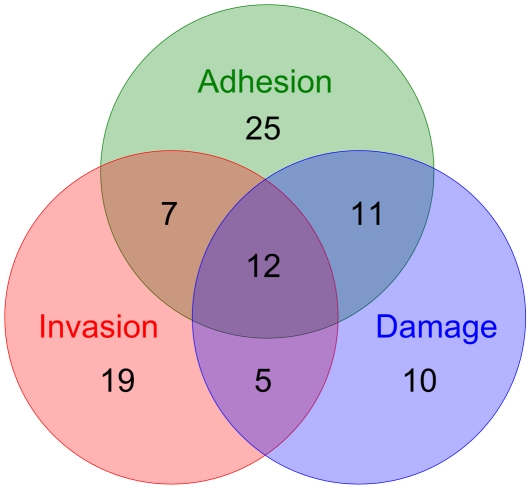
Venn diagram indicating numbers of experimentally validated genes for the three *C. albicans* infection stages. The diagram shows the numbers of overlapping and non-overlapping experimentally validated genes in each infection stage. There are 55, 43, and 38 genes in the adhesion, invasion, and damage stages of *C. albicans* infection, respectively. The complete lists of experimentally validated genes are shown in Table S1.

**Figure 3:**
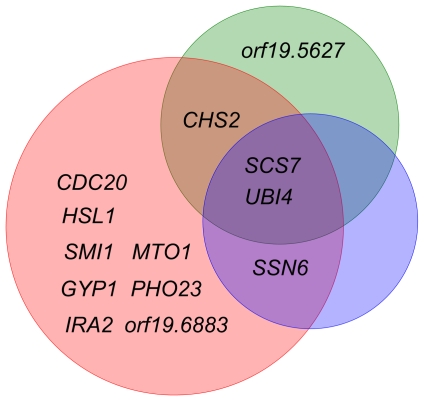
Predicted *C. albicans* infection stage-associated genes. The figure demonstrates this study’s predicted *C. albicans* infection stage-associated genes using a cellular network approach. The green, red, and blue circles indicate the adhesion, invasion, and damage stages of *C. albicans* infection, respectively. There are 4, 12, and 3 genes in each respective stage. The gene names are as listed in the CGD database.

The phenotype-associated gene prediction method was applied to predict *C. albicans* infection-stage associated genes and to investigate *C. albicans*-host interactions in the infection process. Based on the microarray data from Zakikhany *et al.* [5] and some database information, the *C. albicans* gene regulatory and protein interaction networks during infection were constructed [16]. In addition, based on the mutant phenotype information from the CGD and previous literature, three pools of experimentally validated genes, comprising of 55, 43, and 38 genes for the adhesion, invasion, and damage stages of infection respectively, were collected (Figure 2 and Table S1). According to the constructed cellular networks and the experimentally validated gene pools, 4, 12, and 3 genes were predicted as adhesion-, invasion-, and damage-associated genes during *C. albicans* infection (Figure 3). These genes were further investigated to reveal the underlying mechanisms of *C. albicans*-host interactions in the infection process.

### Investigation of *C. albicans* Adhesion Stage-associated Genes

The initial contact of *C. albicans* to host tissues characterizes the first step of *C. albicans* infection. It is a critical step for the establishment of mucosal infection since physical contact to the host cells is sufficient to trigger *C. albicans* hyphal growth and biofilm development, facilitating invasion and damage of host cells [4], [42]. Adhesion is mediated by the interaction between the fungal cell wall and the surface of host cells. As the composition of *C. albicans* cell surface is continually changing, especially during the yeast-to-hyphal transition, adhesion is anticipated as a multi-factorial process [43]. Several proteins have been identified as involved in the adhesion process (Table S1). These include the agglutinin-like sequence (Als) family (e.g., Als3), hyphae-associated proteins (Hwp1), cell wall-associated proteins (Eap1, Ecm33, Mp65, Phr1), secreted proteins (Sap10), and some internal proteins (Rsr1, Big1) [4], [8], [43], [44]. Although deletion of these genes results in decreased adhesion of *C. albicans* to host cells, the mechanisms by which these genes/proteins mediate adhesion are only partially understood. Further, it may be difficult to determine whether these genes/proteins mediate adhesion directly or indirectly, because many of them have complex functions.

In this study, four genes–*CHS2, orf19.5627*, *SCS7*, and *UBI4*–were predicted as adhesion stage-associated genes during *C. albicans* infection (Figure 3). *CHS2* encodes one of the four chitin synthases in *C. albicans*, which catalyze the synthesis of chitin. Chitin is an essential structural polysaccharide in the fungal cell wall that is required for cell shape and morphogenesis [45]. Since adhesion is mediated by the interaction between *C. albicans* cell wall and host cells, it is reasonable to speculate that the synthesis of cell wall components plays a role in adhesion process. In fact, it has been shown that inhibition of chitin synthase activity results in reduced adhesion of *C. albicans* to epithelial cells [46]. Therefore, mutation of *CHS2* may lead to impaired cell wall construction and thus influence *C. albicans* adhesion to host cells. In addition to chitin, other cell wall components may also affect *C. albicans* adhesion. Tsai *et al.* recently identified the human antimicrobial peptide LL-37, which reduces *C. albicans* infectivity by inhibiting adhesion [47]. It has been found that the inhibitory effects of LL-37 on cell adhesion are actualized through interacting with cell wall carbohydrates. Consequently, cell wall components may become potential therapeutic targets for the prevention of *C. albicans* colonization and infection. *SCS7* encodes a putative ceramide hydroxylase and is involved in sphingolipid biosynthesis [48]. Sphingolipids are a class of important membrane lipid components that have been shown to play critical roles in the regulation of several pathobiological processes [49]. However, the roles of sphingolipids in fungal infections are not well characterized since the biological functions of fungal sphingolipids have been studied almost exclusively in nonpathogenic fungi such as *S. cerevisiae* [49]. Although further studies are needed, it has been shown that disruption of sphingolipid synthesis reduces *C. albicans* adhesion [50]. Therefore, *SCS7* may also play some roles in cell adhesion. In addition, several studies have indicated that *SCS7* is down-regulated by iron deprivation [48], [51]. Consequently, iron deficiency may affect the remodeling of membrane lipids and sphingolipid homeostasis [48], thereby having an impact on *C. albicans* infections. The *UBI4* gene encodes polyubiquitin, an ubiquitin precursor protein. Ubiquitination, the addition of ubiquitin to a protein substrate, is a fundamental regulatory post-translational modification event. With the combination of molecular, cellular, and proteomic approaches, Leach *et al.* suggested that ubiquitination contributes to the regulation of several key cellular processes in *C. albicans*, including cell cycle progression, morphogenesis, stress adaptation, and metabolic reprogramming [52]. Further, it has also been shown that *UBI4* inactivation leads to attenuation in virulence of *C. albicans* [52], highlighting the importance of *UBI4* during *C. albicans* infection. Specifically, *UBI4* mutants displayed higher sensitivity to cell wall stress, such as anti-fungal drugs targeting chitin and glucan biosynthesis, indicating that ubiquitination influences cell wall remodeling [52]. As a result, cell adhesion of *C. albicans* may be indirectly regulated by ubiquitination. *orf19.5627* is an uncharacterized gene of unknown function. Further research is needed to examine its relation with adhesion.

### Investigation of *C. albicans* Invasion Stage-associated Genes

During infection, *C. albicans* can utilize two distinct mechanisms to invade host cells: induced endocytosis and active penetration [4], [8], [43]. Since yeast cells do not appear to induce their own uptake into host cells and apparently not penetrate into host cells [7], it is evident that morphogenesis, or yeast-to-hyphal transition, is a critical attribute of *C. albicans* invasion. Induced endocytosis is the process by which a fungal invasin protein interacts with a host surface protein, triggering pseudopod formation and fungal engulfment into the host cell [43]. Two invasins, Als3 and Hsp70, have been identified in *C. albicans* [43]. The endocytosis process is a host actin-dependent as well as host-driven process, as killed fungal hyphae can be endocytosed [4], [7]. Unlike induced endocytosis, active penetration, the other mechanism responsible for *C. albicans* invasion, is fungal-driven and results in hyphal penetration either directly into host cells or at intercellular junctions [4], [43]. However, the process is not well studied, and the question of which particular fungal proteins contribute to the process is still unclear. Further, it should be noted that *C. albicans* invasion also depends on host cell type: while invasion into oral cells occurs via both mechanisms, invasion into intestinal cells occurs only via active penetration [7].

A total of 12 genes were predicted as *C. albicans* invasion stage-associated genes in this study (Figure 3). *CHS2*, *SCS7*, and *UBI4* were predicted as both adhesion and invasion stage-associated genes (*SCS7* and *UBI4* were also identified as damage stage-associated genes). Mutation of *CHS2* has been found to have a profound effect on the chitin content of hyphal cells but not on yeast cells [53], indicating that *CHS2* may be involved in hyphal growth. In addition, the blockage of sphingolipid biosynthesis led to abnormal hyphal morphogenesis [54] and ubiquitination was suggested to regulate morphogenesis in *C. albicans* [52]. Since hyphal growth is crucial for *C. albicans* invasion, *CHS2*, *SCS7*, and *UBI4* may contribute to this process. *CDC20* encodes a protein that is required for the metaphase-to-anaphase transition and mitotic exit during the cell cycle. Depletion of Cdc20 in a mutant strain displaying resulted in highly polarized growth of yeast buds under yeast growth conditions but had no influence on serum-induced hyphal growth [55]. Further studies are required to investigate the association between Cdc20-mediated cell cycle progression and cell invasion. *HSL1* encodes a protein kinase that has been shown to play a role in the suppression of cell elongation. *HSL1* knockout showed an elongated cell phenotype in both yeast- and hyphae-inducing media [56]. We thus may speculate that mutation of *HSL1* would promote the cell invasion ability during infection due to enhanced hyphal growth. *SMI1* encodes a regulator of glucan synthesis. Mutation of *SMI1* affected biofilm matrix and cell wall β-1,3-glucan production [57], which further influenced biofilm-associated drug resistance mechanisms. Kitamura *et al.* have revealed that inhibitors of β-1,6-glucan reduce hyphal elongation during the *C. albicans* invasion process [58], suggesting a role of *SMI1* in *C. albicans* invasion. *SSN6* encodes a putative transcriptional regulator. Deletion of *SSN6* resulted in defective hyphal development while overexpression of *SSN6* led to enhanced filamentous growth [59], indicating that Ssn6 regulates filamentous growth and thus may regulate cell invasion. *GYP1*, *IRA2*, *MTO1*, *PHO23*, and *orf19.6883* are all uncharacterized genes predicted as *C. albicans* invasion stage-associated genes in this study. Although their functions need to be further characterized, it is likely that they contribute to host cell invasion either directly or indirectly.

### Investigation of *C. albicans* Damage Stage-associated Genes

The last stage of *C. albicans* infection is characterized by substantial cell damage and destruction of host tissues. However, the mechanism by which *C. albicans* induces host cell damage is poorly understood. It was originally thought that host cell invasion would induce cell damage. Nevertheless, experiments have indicated that some *C. albicans* mutant strains with normal adhesion and endocytosis are unable to induce cell damage [6]. Therefore, active penetration appears to be more essential for the induction of cell damage, yet the hypothesis remains to be validated experimentally. Numerous factors, such as hyphal formation and secreted lytic enzymes [8], [43] have been suggested as contributors to host cell damage, but more than just these factors are likely required for tissue destruction. Other fungal factors required for cell damage are as yet unidentified.

*SSN6*, *SCS7*, and *UBI4* were predicted as *C. albicans* damage stage-associated genes in this study (Figure 3). Since *SSN6* encodes a putative transcriptional regulator, it may affect cell damage indirectly by regulating damage-associated genes. *SCS7* and *UBI4* were identified as genes associated with adhesion, invasion, and damage. Although there is no clear evidence indicating how sphingolipid biosynthesis and ubiquitination induce host cell damage during *C. albicans* infection, these two cellular processes are worth further investigation.

## Discussion

*Candida albicans* infection has emerged as significant cause of mortality in humans. Although several attributes have been associated with *C. albicans* pathogenesis and *C. albicans* infection has been recognized as a complex process, understanding of the underlying molecular mechanisms remains relatively limited. Due to the complexity of the host-pathogen interaction, systems biology approaches are more suitable for investigating the infection process. Unlike traditional biological research, which has intensely focused on individual components (genes/proteins) involved in biological processes, systems biology addresses biological phenomena from the systems perspective [60], [61]. By integration of high-throughput omics data, mathematical models can be constructed to describe the interactions between the biological components of a complex system. Through model development and system analysis, critical components of a system, such as the complex host-pathogen system, can be discovered. We previously developed a network comparison framework and identified 23 potential transcription factors controlling *C. albicans* biofilm formation [20]. Further experiments have shown that mutations in some identified genes result in alteration of biofilm formation (unpublished data), validating the proposed systems biology method. In this study, in light of the principles that the proteins that lie closer to one another in the PPI network are more likely to have similar functions, and that genes regulated by the same transcription factors tend to have similar functions, a cellular network approach was proposed to predict phenotype-associated genes that are responsible for the three infection stages. A total of 4, 12, and 3 genes were predicted as adhesion-, invasion-, and damage-associated genes during *C. albicans* infection. Based on the results, we found that the predicted adhesion stage-associated genes all contribute to the regulation or synthesis of cell surface components. Consequently, we concluded that the cell surface components and their related proteins play pivotal roles in cell adhesion. In fact, the targets of existing antifungal agents are mainly located on the cell surface [62]. Therefore, the newly predicted genes may become promising therapeutic candidates or provide guidance for the discovery of novel therapies. In addition, based on the predicted invasion stage-associated genes, morphogenesis emerged as a critical feature for cell invasion. All predicted genes are associated with morphogenesis directly or indirectly. Although morphogenesis is not necessarily a pathogenicity determinant, it is definitely a key characteristic of host cell invasion. Therefore, pathways that transduce hyphae-inducing signals and regulate downstream transcription factors may be potential candidates for characterizing cell invasion mechanisms and targeted inhibition of the yeast-to-hyphal transition may be attractive options for controlling *C. albicans* infection [63]. Unlike the adhesion and invasion processes, where mechanisms are partially understood, little is known about the damage mechanism during *C. albicans* infection. If more genes can be experimentally characterized, more damage stage-associated genes could be predicted. Consequently, we might be able to better infer the damage mechanism during *C. albicans* infection.

Although there are distinctive characteristics for the three stages of *C. albicans* infection, these processes are not necessarily mutually exclusive and are likely to involve significant overlap in function [43]. Based on the experimentally validated and predicted genes examined in this study (Figure 2, Figure 3, and Table S1), we also found that several genes contribute to more than one stage of *C. albicans* infection. A previous study has indicated that the core elements of the cAMP-PKA pathway are required for all stages of infection [6]. We further speculate that *SCS7* and *UBI4* may be involved in the cAMP-PKA pathway as well since they are predicted to be important in all three stages according to our results. On the other hand, some genes were predicted or verified as stage-specific genes, i.e., they only contribute to one stage of *C. albicans* infection. These stage-specific genes can be used to identify factors specifically involved in a certain infection stage. Using the cellular network approach to predict phenotype-associated genes is not only useful for the investigation of *C. albicans* infection, it can also be employed under different experimental conditions and in different organisms, if the required data are available and provided. While our approach has been shown to be useful, some improvements can be made. An apparent limitation is that some data may be acquired from other organisms different from the organism of interest. In this study, due to a lack of sufficient information in *C. albicans* protein-protein interactions, such information was completely inferred from the model organism *S*. *cerevisiae* with the help of ortholog mapping data. Comparative genomics to *S. cerevisiae* provides an alternative way to infer potential protein-protein interactions in *C. albicans*. However, using information derived from *S*. *cerevisiae* may lead to misinterpretation. Recently, many studies have focused on the development of protein-protein interaction assay tools in *C. albicans*. For instance, Boysen *et al.* developed a vesicle targeting method to detect PPIs in *C. albicans* [64]. Stynen *et al.* constructed a functional *C. albicans* two-hybrid system [65]. With these tools developed, we can expect protein-protein interactions to be massively screened in the near future. Once reliable information regarding *C. albicans* protein-protein interactions becomes available, more accurate cellular networks can be constructed, more significant phenotype-associated genes can be predicted, and so mechanisms of the three *C. albicans* infection stages can be further investigated. This study has emphasized more heavily the pathogen aspect in the host-pathogen interactions during *C. albicans* infection. However, to better comprehend the interactions between the pathogen and the host, it is essential to understand the host defense processes combating the pathogen, especially immune responses. Consequently, if the defensive and invasive mechanisms of such host-pathogen interactions could be elucidated simultaneously, that comprehensive knowledge would be instrumental in the identification of novel therapies to treat *C. albicans* infection.

## Supporting Information

Text S1 **Details of cellular network construction.**
(PDF)

Table S1 **Experimentally validated genes for the three** ***C. albicans*** **infection stages.**
(PDF)
