# Supplementary material for: Prediction of Phenotype-Associated Genes via a Cellular Network Approach: A Candida albicans Infection Case Study
Source: PLoS One. 2012 Apr 11;7(4):e35339. doi: 10.1371/journal.pone.0035339 (PMC3324557; doi:10.1371/journal.pone.0035339)
Supplement: Text S1 — Details of cellular network construction. (PDF) [file pone.0035339.s001.pdf]

Text S1. Details of cellular network construction

For a target gene i in the candidate gene regulatory network, the dynamic transcriptional regulatory model of the gene expression was described by the following equation [1]:

$$x_i[t+1] = x_i[t] + \sum_{j=1}^{N_i} a_{ij} z_j[t] - \lambda_i x_i[t] + k_i + \varepsilon_i[t] \tag{S1}$$

where $x_i[t]$ represents the gene expression level at time t for target gene i, a_{ij} denotes the regulatory ability of j-th transcription factor (TF) toward i-th target gene, $z_j[t]$ represents the regulation function of j-th TF, λ_i indicates the mRNA degradation effect, k_i represents the basal level, $\varepsilon_i[t]$ denotes the stochastic noise due to the model uncertainty and the fluctuation of microarray data. The regulation function $z_j[t]$ can be modeled as the sigmoid function of $y_j[t]$ (the protein expression of transcription factor j) [2,3]:

$$z_j[t] = f_j(y_j[t]) = \frac{1}{1+\exp\left\{-(y_j[t]-\mu_j)/\sigma_j\right\}} \tag{S2}$$

where f_j denotes the sigmoid function, μ_j and σ_j represent the mean and standard deviation of protein expression level of TF j.

Similarly, for a target protein n in the candidate protein interaction network, the dynamic model of the protein expression was as follows [1]:

$$y_n[t+1] = y_n[t] + \sum_{m=1}^{M_n} b_{nm} y_n[t] y_m[t] + \alpha_n x_n[t] - \beta_n y_n[t] + h_n + \omega_n[t] \tag{S3}$$

where $y_n[t]$ represents the protein expression level at time t of target protein n, b_{nm} denotes the interaction ability of m-th interactive protein to n-th target protein, $y_m[t]$ represents the protein expression level of m-th protein interacting with target protein n, α_n denotes the translation effect from mRNA to protein, $x_n[t]$ represents the

mRNA expression level of the corresponding target protein *n*, β_n indicates the protein degradation effect, h_n represents the basal expression level, and $\omega_n[t]$ is the stochastic noise.

After the dynamic models of candidate gene regulatory network and candidate protein interaction network were built, the regulatory/interaction parameters in the models have to be identified with the help of time-course microarray data. The strategy was to identify the network parameters gene by gene (and protein by protein) by solving a constrained least square parameter estimation problem. Equation (S1) can be rewritten as the following regression form:

$$\begin{aligned} x_i[t+1] &= \begin{bmatrix} z_1[t] & \cdots & z_{N_i}[t] & x_i[t] & 1 \end{bmatrix} \cdot \begin{bmatrix} a_{i1} \\ \vdots \\ a_{iN_i} \\ (1-\lambda_i) \\ k_i \end{bmatrix} + \varepsilon_i[t] \\ &\equiv \phi_i[t] \cdot \theta_i + \varepsilon_i[t] \end{aligned} \tag{S4}$$

where $\phi_i[t]$ denotes the regression vector which can be obtained from the processing above. θ_i is the parameter vector of the target gene *i* which is to be estimated. In order to avoid overfitting when identifying the regulatory parameters, the cubic spline method was also used to interpolate extra time points for gene expression data. By the cubic spline method, we can easily get the values of $\{z_j[t_l] \quad x_i[t_l]\}$ for $l \in \{1,\ 2,\ \cdots, L\}$ and $j \in \{1,\ 2,\ \cdots, N_i\}$, where L is the number of expression time points of a target gene *i*, and N_i is the number of TFs binding to the target gene *i*. Equation (S4) at different time points can be arranged as follows:

$$\begin{bmatrix} x_i[t_2] \\ x_i[t_3] \\ \vdots \\ x_i[t_L] \end{bmatrix} = \begin{bmatrix} \phi_i[t_1] \\ \phi_i[t_2] \\ \vdots \\ \phi_i[t_{L-1}] \end{bmatrix} \cdot \theta_i + \begin{bmatrix} \varepsilon_i[t_1] \\ \varepsilon_i[t_2] \\ \vdots \\ \varepsilon_i[t_{L-1}] \end{bmatrix} \tag{S5}$$

For simplicity, the notations X_i, Φ_i, and E_i were defined to represent equation (S5) as follows:

$$X_i = \Phi_i \cdot \theta_i + \mathrm{E}_i \tag{S6}$$

The constrained least square parameter estimation problem was formulated as follows:

$$\min_{\theta_i} \frac{1}{2} \left\| \Phi_i \theta_i - X_i \right\|_2^2 \quad \text{such that } A\theta_i \leq b \tag{S7}$$

where $A = [0 \ \cdots \ 0 \ \ 0 \ \ -1]$, $b = 0$ give the constraints to force the basal level k_i in equation (S1) to be always non-negative, i.e., $k_i \geq 0$. The constrained least square problem can be solved using the active set method for quadratic programming [4].

Similarly, equation (S3) can be rewritten in the following regression form:

$$y_n[t+1] = \left[y_n[t] y_1[t] \ \cdots \ y_n[t] y_{M_n}[t] \ \ x_n[t] \ \ y_n[t] \ \ 1 \right] \cdot \begin{bmatrix} b_{n1} \\ \vdots \\ b_{nM_n} \\ \alpha_n \\ (1-\beta_n) \\ h_n \end{bmatrix} + \omega_n[t] \tag{S8}$$

$$\equiv \psi_n[t] \cdot \eta_n + \omega_n[t]$$

where $\psi_n[t]$ indicates the regression vector and η_n is the parameter vector to be estimated. By cubic spline method, at different time points, equation (S8) can be presented as the following equation:

$$Y_n = \Psi_n \cdot \eta_n + \Omega_n \tag{S9}$$

The identification problem was then formulated as follows:

$$\min_{\eta_n} \frac{1}{2} \left\| \Psi_n \eta_n - Y_n \right\|_2^2 \quad \text{such that } C\eta_n \le d \tag{S10}$$

where $C = \text{diag}[0 \ \cdots \ 0 \ -1 \ 0 \ -1]$ and $d = [0 \ \cdots \ 0]^T$, indicating that the translation effect α_n and the basal expression level h_n are non-negative. Since there are no good data available for genome-wide protein expression levels in *C. albicans*, mRNA expression profiles were used to substitute for the protein expression levels when identifying the interaction parameters.

Once the regulatory abilities a_{ij} and interaction abilities b_{nm} were estimated, the Akaike Information Criterion (AIC) [5,6] was applied to detect the significant regulations and interactions in candidate gene regulatory network and protein interaction network. AIC, which includes both estimated residual error and model complexity in one statistics, quantifies the relative goodness of fit of a model. For a transcriptional regulatory model with N_i regulatory parameters (or TFs) to fit with data from L samples, the AIC can be written as follows [5,6]:

$$\text{AIC}(N_i) = \log\left(\frac{1}{L} \left(X_i - \hat{X}_i \right)^T \left(X_i - \hat{X}_i \right) \right) + \frac{2N_i}{L} \tag{S11}$$

where $\hat{X}_i$ denotes the estimated expression profile of the i-th target gene, i.e. $\hat{X}_i = \Phi_i \cdot \hat{\theta}_i$, and $\hat{\sigma}_i^2 = \frac{1}{L}\left(X_i - \hat{X}_i \right)^T \left(X_i - \hat{X}_i \right)$ is the estimated residual error. As the residual error $\hat{\sigma}_i^2$ decreases, the AIC decreases. In contrast, while the number of regulatory TFs (or parameters) N_i increases, the AIC increases. Therefore, there is a tradeoff between residual error and model order. As the expected residual error decreases with increasing regulatory TF numbers in models of inadequate complexity, there should be a minimum around the optimal regulatory TF number. The minimization achieved in equation (S11) will indicate the ideal model order (i.e. the optimal number of TFs that regulate the target gene) of the transcriptional regulatory system. With the statistical selection of N_i regulatory TFs by minimization of the AIC,

the question of whether a regulatory TF is a significant one or just a false positive for the *i*-th target gene can be determined. In this way, the candidate gene regulatory network was refined and the significant gene regulatory network for *C. albicans* infection was constructed. Similarly, the significant protein interaction network for *C. albicans* infection can also be constructed with AIC.

References

1. Wang YC, Chen BS (2010) Integrated cellular network of transcription regulations and protein-protein interactions. BMC Syst Biol 4: 20.
2. Chang YH, Wang YC, Chen BS (2006) Identification of transcription factor cooperativity via stochastic system model. Bioinformatics 22: 2276-2282.
3. Alon U (2007) An introduction to systems biology : design principles of biological circuits. Boca Raton, FL: Chapman & Hall/CRC. xvi, 301 p., 304 p. of plates p.
4. Gill PE, Murray W, Wright MH (1981) Practical optimization. London ; New York: Academic Press. xvi, 401 p. p.
5. Johansson R (1993) System modeling and identification. Englewood Cliffs, NJ: Prentice Hall.
6. Akaike H (1974) New Look at Statistical-Model Identification. IEEE Transactions on Automatic Control Ac19: 716-723.
