# Supplementary material for: Prediction of Phenotype-Associated Genes via a Cellular Network Approach: A Candida albicans Infection Case Study
Source: PLoS One. 2012 Apr 11;7(4):e35339. doi: 10.1371/journal.pone.0035339 (PMC3324557; doi:10.1371/journal.pone.0035339)
Supplement: Table S1 — Experimentally validated genes for the three C. albicans infection stages. (PDF) [file pone.0035339.s002.pdf]

Table S1. Experimentally validated genes for the three *C. albicans* infection stages

Gene	orf	Adhesion	Invasion	Damage	Reference
AHR1	*orf19.7381*	V			[1]
ALS1	*orf19.5741*	V	V		[2,3]
ALS2	*orf19.1097*	V		V	[4]
ALS3	*orf19.1816*	V	V	V	[5]
ALS4	*orf19.4555*	V			[4]
ALS9	*orf19.5742*	V			[6]
ASC1	*orf19.6906*	V	V		[7]
BCR1	*orf19.723*	V		V	[5]
BIG1	*orf19.2334*	V			[8]
BMH1	*orf19.3014*		V		[9]
BUD2	*orf19.940*	V		V	[5]
CDC10	*orf19.548*		V		[10]
CDC11	*orf19.5691*		V		[10]
CDC14	*orf19.4192*		V		[11]
CKA2	*orf19.3530*	V		V	[5]
CSH1	*orf19.4477*	V			[12]
CZF1	*orf19.3127*	V	V	V	[5]
DCK1	*orf19.815*		V		[13]
DEF1	*orf19.7561*			V	[5]
DFG16	*orf19.881*		V		[14]
DFI1	*orf19.7084*	V	V		[15]
DRG1	*orf19.5083*		V		[16]
EAP1	*orf19.1401*	V			[17]
ECM33	*orf19.3010.1*	V	V	V	[5]
EFG1	*orf19.610*	V	V	V	[5]
FET3	*orf19.4211*	V			[18]
FTR1	*orf19.7219*			V	[19]
GPA2	*orf19.1621*		V		[20]
GPD2	*orf19.691*			V	[5]
GPR1	*orf19.1944*		V		[21]
GUP1	*orf19.4985*	V	V		[22]
HDA1	*orf19.2606*		V		[23]
HGC1	*orf19.6028*	V	V	V	[5]

HIS4	*orf19.5639*	V			[24]
HSP70	*orf19.4980*		V	V	[25]
HWP1	*orf19.1321*	V		V	[5]
HWP2	*orf19.3380*	V			[26]
ICL1	*orf19.6844*		V	V	[5]
INT1	*orf19.4257*	V			[27]
IPT1	*orf19.4769*	V			[28]
IRS4	*orf19.6953*	V			[29]
KEX2	*orf19.4755*		V		[30]
KRE5	*orf19.290*	V			[31]
LMO1	*orf19.5147*		V		[32]
MKC1	*orf19.7523*			V	[5]
MNT1	*orf19.1665*	V			[33]
MNT2	*orf19.1663*	V			[33]
MP65	*orf19.1779*	V			[34]
PDE2	*orf19.2972*	V	V		[35]
PEP7	*orf19.5662*	V			[36]
PGA1	*orf19.7625*	V			[37]
PGA34	*orf19.2833*			V	[5]
PHR1	*orf19.3829*	V	V		[38,39]
PLD1	*orf19.1161*		V		[40]
PMT1	*orf19.5171*	V		V	[41,42]
PMT2	*orf19.6812*		V	V	[5]
PMT4	*orf19.4109*			V	[42]
PMT6	*orf19.3802*	V		V	[42,43]
PRA1	*orf19.3111*	V			[44]
RAC1	*orf19.6237*		V		[45]
RAS1	*orf19.1760*	V	V	V	[5]
RHD3	*orf19.5305*			V	[46]
RHR2	*orf19.5437*			V	[5]
RIM101	*orf19.7247*	V	V	V	[5]
RIM13	*orf19.3995*		V		[39]
RIM20	*orf19.4800*		V		[39]
RIM8	*orf19.6091*		V		[39]
RSR1	*orf19.2614*	V		V	[5]
SAP1	*orf19.5714*	V		V	[47,48]
SAP10	*orf19.3839*	V		V	[49]

SAP2	*orf19.3708*	V		V	[47,48]
SAP3	*orf19.6001*	V			[47]
SAP9	*orf19.6928*			V	[49]
SET1	*orf19.6009*	V			[50]
SFL2	*orf19.3969*		V	V	[51,52]
SHE3	*orf19.5595*		V	V	[53]
SIT1	*orf19.2179*		V		[54]
STE2	*orf19.696*	V			[55]
SUN41	*orf19.3642*	V			[56]
SUR7	*orf19.3414*		V		[57]
TEC1	*orf19.5908*	V	V	V	[5]
TPK1	*orf19.4892*	V	V	V	[5]
TPK2	*orf19.2277*	V	V	V	[5]
TPS1	*orf19.6640*	V			[58]
TUP1	*orf19.6109*	V	V	V	[5]
UTR2	*orf19.1671*	V	V		[59]
VPS11	*orf19.4403*	V	V	V	[5]
VPS34	*orf19.6243*	V			[60]
YCK2	*orf19.7001*			V	[61]

References

1. Askew C, Sellam A, Epp E, Mallick J, Hogues H, et al. (2011) The zinc cluster transcription factor Ahr1p directs Mcm1p regulation of Candida albicans adhesion. Mol Microbiol 79: 940-953.

2. Fu Y, Ibrahim AS, Sheppard DC, Chen YC, French SW, et al. (2002) Candida albicans Als1p: an adhesin that is a downstream effector of the EFG1 filamentation pathway. Mol Microbiol 44: 61-72.

3. Phan QT, Myers CL, Fu Y, Sheppard DC, Yeaman MR, et al. (2007) Als3 is a Candida albicans invasin that binds to cadherins and induces endocytosis by host cells. PLoS Biol 5: e64.

4. Zhao X, Oh SH, Yeater KM, Hoyer LL (2005) Analysis of the Candida albicans Als2p and Als4p adhesins suggests the potential for compensatory function within the Als family. Microbiology 151: 1619-1630.

5. Wachtler B, Wilson D, Haedicke K, Dalle F, Hube B (2011) From attachment to damage: defined genes of Candida albicans mediate adhesion, invasion and damage during interaction with oral epithelial cells. PLoS One 6: e17046.

6. Zhao X, Oh SH, Hoyer LL (2007) Unequal contribution of ALS9 alleles to adhesion between Candida albicans and human vascular endothelial cells. Microbiology 153: 2342-2350.

7. Kim SW, Joo YJ, Kim J (2010) Asc1p, a ribosomal protein, plays a pivotal role in cellular adhesion and virulence in Candida albicans. J Microbiol 48: 842-848.

8. Umeyama T, Kaneko A, Watanabe H, Hirai A, Uehara Y, et al. (2006) Deletion of the CaBIG1 gene

reduces beta-1,6-glucan synthesis, filamentation, adhesion, and virulence in Candida albicans. Infect Immun 74: 2373-2381.

9. Palmer GE, Sturtevant JE (2004) Random mutagenesis of an essential Candida albicans gene. Curr Genet 46: 343-356.
10. Warenda AJ, Kauffman S, Sherrill TP, Becker JM, Konopka JB (2003) Candida albicans septin mutants are defective for invasive growth and virulence. Infect Immun 71: 4045-4051.
11. Clemente-Blanco A, Gonzalez-Novo A, Machin F, Caballero-Lima D, Aragon L, et al. (2006) The Cdc14p phosphatase affects late cell-cycle events and morphogenesis in Candida albicans. J Cell Sci 119: 1130-1143.
12. Singleton DR, Masuoka J, Hazen KC (2001) Cloning and analysis of a Candida albicans gene that affects cell surface hydrophobicity. J Bacteriol 183: 3582-3588.
13. Hope H, Bogliolo S, Arkowitz RA, Bassilana M (2008) Activation of Rac1 by the guanine nucleotide exchange factor Dck1 is required for invasive filamentous growth in the pathogen Candida albicans. Mol Biol Cell 19: 3638-3651.
14. Thewes S, Kretschmar M, Park H, Schaller M, Filler SG, et al. (2007) In vivo and ex vivo comparative transcriptional profiling of invasive and non-invasive Candida albicans isolates identifies genes associated with tissue invasion. Mol Microbiol 63: 1606-1628.
15. Zucchi PC, Davis TR, Kumamoto CA (2010) A Candida albicans cell wall-linked protein promotes invasive filamentation into semi-solid medium. Mol Microbiol 76: 733-748.
16. Chen X, Kumamoto CA (2006) A conserved G protein (Drg1p) plays a role in regulation of invasive filamentation in Candida albicans. Microbiology 152: 3691-3700.
17. Li F, Svarovsky MJ, Karlsson AJ, Wagner JP, Marchillo K, et al. (2007) Eap1p, an adhesin that mediates Candida albicans biofilm formation in vitro and in vivo. Eukaryot Cell 6: 931-939.
18. Eck R, Hundt S, Hartl A, Roemer E, Kunkel W (1999) A multicopper oxidase gene from Candida albicans: cloning, characterization and disruption. Microbiology 145 (Pt 9): 2415-2422.
19. Almeida RS, Brunke S, Albrecht A, Thewes S, Laue M, et al. (2008) the hyphal-associated adhesin and invasin Als3 of Candida albicans mediates iron acquisition from host ferritin. PLoS Pathog 4: e1000217.
20. Miwa T, Takagi Y, Shinozaki M, Yun CW, Schell WA, et al. (2004) Gpr1, a putative G-protein-coupled receptor, regulates morphogenesis and hypha formation in the pathogenic fungus Candida albicans. Eukaryot Cell 3: 919-931.
21. Maidan MM, De Rop L, Serneels J, Exler S, Rupp S, et al. (2005) The G protein-coupled receptor Gpr1 and the Galpha protein Gpa2 act through the cAMP-protein kinase A pathway to induce morphogenesis in Candida albicans. Mol Biol Cell 16: 1971-1986.
22. Ferreira C, Silva S, Faria-Oliveira F, Pinho E, Henriques M, et al. (2010) Candida albicans virulence and drug-resistance requires the O-acyltransferase Gup1p. BMC Microbiol 10: 238.
23. Zacchi LF, Schulz WL, Davis DA (2010) HOS2 and HDA1 encode histone deacetylases with opposing roles in Candida albicans morphogenesis. PLoS One 5: e12171.

24. Arie ZR, Altboum Z, Sandovsky-Losica H, Segal E (1998) Adhesion of Candida albicans mutant strains to host tissue. FEMS Microbiol Lett 163: 121-127.
25. Sun JN, Solis NV, Phan QT, Bajwa JS, Kashleva H, et al. (2010) Host cell invasion and virulence mediated by Candida albicans Ssa1. PLoS Pathog 6: e1001181.
26. Younes S, Bahnan W, Dimassi HI, Khalaf RA (2011) The Candida albicans Hwp2 is necessary for proper adhesion, biofilm formation and oxidative stress tolerance. Microbiol Res 166: 430-436.
27. Gale CA, Bendel CM, McClellan M, Hauser M, Becker JM, et al. (1998) Linkage of adhesion, filamentous growth, and virulence in Candida albicans to a single gene, INT1. Science 279: 1355-1358.
28. Rouabhia M, Mukherjee PK, Lattif AA, Curt S, Chandra J, et al. (2011) Disruption of sphingolipid biosynthetic gene IPT1 reduces Candida albicans adhesion and prevents activation of human gingival epithelial cell innate immune defense. Med Mycol 49: 458-466.
29. Badrane H, Cheng S, Nguyen MH, Jia HY, Zhang Z, et al. (2005) Candida albicans IRS4 contributes to hyphal formation and virulence after the initial stages of disseminated candidiasis. Microbiology 151: 2923-2931.
30. Newport G, Kuo A, Flattery A, Gill C, Blake JJ, et al. (2003) Inactivation of Kex2p diminishes the virulence of Candida albicans. J Biol Chem 278: 1713-1720.
31. Herrero AB, Magnelli P, Mansour MK, Levitz SM, Bussey H, et al. (2004) KRE5 gene null mutant strains of Candida albicans are avirulent and have altered cell wall composition and hypha formation properties. Eukaryot Cell 3: 1423-1432.
32. Hope H, Schmauch C, Arkowitz RA, Bassilana M (2010) The Candida albicans ELMO homologue functions together with Rac1 and Dck1, upstream of the MAP Kinase Cek1, in invasive filamentous growth. Mol Microbiol 76: 1572-1590.
33. Munro CA, Bates S, Buurman ET, Hughes HB, Maccallum DM, et al. (2005) Mnt1p and Mnt2p of Candida albicans are partially redundant alpha-1,2-mannosyltransferases that participate in O-linked mannosylation and are required for adhesion and virulence. J Biol Chem 280: 1051-1060.
34. Sandini S, Stringaro A, Arancia S, Colone M, Mondello F, et al. (2011) The MP65 gene is required for cell wall integrity, adherence to epithelial cells and biofilm formation in Candida albicans. BMC Microbiol 11: 106.
35. Wilson D, Tutulan-Cunita A, Jung W, Hauser NC, Hernandez R, et al. (2007) Deletion of the high-affinity cAMP phosphodiesterase encoded by PDE2 affects stress responses and virulence in Candida albicans. Mol Microbiol 65: 841-856.
36. Franke K, Nguyen M, Hartl A, Dahse HM, Vogl G, et al. (2006) The vesicle transport protein Vac1p is required for virulence of Candida albicans. Microbiology 152: 3111-3121.
37. Hashash R, Younes S, Bahnan W, El Koussa J, Maalouf K, et al. (2011) Characterisation of Pga1, a putative Candida albicans cell wall protein necessary for proper adhesion and biofilm formation. Mycoses 54: 491-500.
38. Calderon J, Zavrel M, Ragni E, Fonzi WA, Rupp S, et al. (2010) PHR1, a pH-regulated gene of

Candida albicans encoding a glucan-remodelling enzyme, is required for adhesion and invasion. Microbiology 156: 2484-2494.

39. Yuan X, Mitchell BM, Hua X, Davis DA, Wilhelmus KR (2010) The RIM101 signal transduction pathway regulates Candida albicans virulence during experimental keratomycosis. Invest Ophthalmol Vis Sci 51: 4668-4676.
40. Hube B, Hess D, Baker CA, Schaller M, Schafer W, et al. (2001) The role and relevance of phospholipase D1 during growth and dimorphism of Candida albicans. Microbiology 147: 879-889.
41. Timpel C, Strahl-Bolsinger S, Ziegelbauer K, Ernst JF (1998) Multiple functions of Pmt1p-mediated protein O-mannosylation in the fungal pathogen Candida albicans. J Biol Chem 273: 20837-20846.
42. Rouabhia M, Schaller M, Corbucci C, Vecchiarelli A, Prill SK, et al. (2005) Virulence of the fungal pathogen Candida albicans requires the five isoforms of protein mannosyltransferases. Infect Immun 73: 4571-4580.
43. Timpel C, Zink S, Strahl-Bolsinger S, Schroppel K, Ernst J (2000) Morphogenesis, adhesive properties, and antifungal resistance depend on the Pmt6 protein mannosyltransferase in the fungal pathogen candida albicans. J Bacteriol 182: 3063-3071.
44. Soloviev DA, Fonzi WA, Sentandreu R, Pluskota E, Forsyth CB, et al. (2007) Identification of pH-regulated antigen 1 released from Candida albicans as the major ligand for leukocyte integrin alphaMbeta2. J Immunol 178: 2038-2046.
45. Bassilana M, Arkowitz RA (2006) Rac1 and Cdc42 have different roles in Candida albicans development. Eukaryot Cell 5: 321-329.
46. de Boer AD, de Groot PW, Weindl G, Schaller M, Riedel D, et al. (2010) The Candida albicans cell wall protein Rhd3/Pga29 is abundant in the yeast form and contributes to virulence. Yeast 27: 611-624.
47. Watts HJ, Cheah FS, Hube B, Sanglard D, Gow NA (1998) Altered adherence in strains of Candida albicans harbouring null mutations in secreted aspartic proteinase genes. FEMS Microbiol Lett 159: 129-135.
48. Schaller M, Bein M, Korting HC, Baur S, Hamm G, et al. (2003) The secreted aspartyl proteinases Sap1 and Sap2 cause tissue damage in an in vitro model of vaginal candidiasis based on reconstituted human vaginal epithelium. Infect Immun 71: 3227-3234.
49. Albrecht A, Felk A, Pichova I, Naglik JR, Schaller M, et al. (2006) Glycosylphosphatidylinositol-anchored proteases of Candida albicans target proteins necessary for both cellular processes and host-pathogen interactions. J Biol Chem 281: 688-694.
50. Raman SB, Nguyen MH, Zhang Z, Cheng S, Jia HY, et al. (2006) Candida albicans SET1 encodes a histone 3 lysine 4 methyltransferase that contributes to the pathogenesis of invasive candidiasis. Mol Microbiol 60: 697-709.
51. Song W, Wang H, Chen J (2011) Candida albicans Sfl2, a temperature-induced transcriptional regulator, is required for virulence in a murine gastrointestinal infection model. FEMS Yeast Res 11:

209-222.

52. Spiering MJ, Moran GP, Chauvel M, Maccallum DM, Higgins J, et al. (2010) Comparative transcript profiling of Candida albicans and Candida dubliniensis identifies SFL2, a C. albicans gene required for virulence in a reconstituted epithelial infection model. Eukaryot Cell 9: 251-265.
53. Elson SL, Noble SM, Solis NV, Filler SG, Johnson AD (2009) An RNA transport system in Candida albicans regulates hyphal morphology and invasive growth. PLoS Genet 5: e1000664.
54. Heymann P, Gerads M, Schaller M, Dromer F, Winkelmann G, et al. (2002) The siderophore iron transporter of Candida albicans (Sit1p/Arn1p) mediates uptake of ferrichrome-type siderophores and is required for epithelial invasion. Infect Immun 70: 5246-5255.
55. Yi S, Sahni N, Pujol C, Daniels KJ, Srikantha T, et al. (2009) A Candida albicans-specific region of the alpha-pheromone receptor plays a selective role in the white cell pheromone response. Mol Microbiol 71: 925-947.
56. Hiller E, Heine S, Brunner H, Rupp S (2007) Candida albicans Sun41p, a putative glycosidase, is involved in morphogenesis, cell wall biogenesis, and biofilm formation. Eukaryot Cell 6: 2056-2065.
57. Alvarez FJ, Douglas LM, Rosebrock A, Konopka JB (2008) The Sur7 protein regulates plasma membrane organization and prevents intracellular cell wall growth in Candida albicans. Mol Biol Cell 19: 5214-5225.
58. Martinez-Esparza M, Aguinaga A, Gonzalez-Parraga P, Garcia-Penarrubia P, Jouault T, et al. (2007) Role of trehalose in resistance to macrophage killing: study with a tps1/tps1 trehalose-deficient mutant of Candida albicans. Clin Microbiol Infect 13: 384-394.
59. Alberti-Segui C, Morales AJ, Xing H, Kessler MM, Willins DA, et al. (2004) Identification of potential cell-surface proteins in Candida albicans and investigation of the role of a putative cell-surface glycosidase in adhesion and virulence. Yeast 21: 285-302.
60. Bruckmann A, Kunkel W, Hartl A, Wetzker R, Eck R (2000) A phosphatidylinositol 3-kinase of Candida albicans influences adhesion, filamentous growth and virulence. Microbiology 146 (Pt 11): 2755-2764.
61. Park H, Liu Y, Solis N, Spotkov J, Hamaker J, et al. (2009) Transcriptional responses of candida albicans to epithelial and endothelial cells. Eukaryot Cell 8: 1498-1510.
